# Stores, Channels, Glue, and Trees: Active Glial and Active Dendritic Physiology

**DOI:** 10.1007/s12035-018-1223-5

**Published:** 2018-07-16

**Authors:** Sufyan Ashhad, Rishikesh Narayanan

**Affiliations:** 1 Department of Neurobiology, University of California at Los Angeles, Los Angeles, CA 90095 USA; 2 Cellular Neurophysiology Laboratory, Molecular Biophysics Unit, Indian Institute of Science, Bangalore, 560012 India

**Keywords:** Endoplasmic reticulum, Ion channels, Astrocytes, Active dendrites, Gliotransmission

## Abstract

Glial cells and neuronal dendrites were historically assumed to be passive structures that play only supportive physiological roles, with no active contribution to information processing in the central nervous system. Research spanning the past few decades has clearly established this assumption to be far from physiological realities. Whereas the discovery of active channel conductances and their localized plasticity was the turning point for dendritic structures, the demonstration that glial cells release transmitter molecules and communicate across the neuroglia syncytium through calcium wave propagation constituted path-breaking discoveries for glial cell physiology. An additional commonality between these two structures is the ability of calcium stores within their endoplasmic reticulum (ER) to support active propagation of calcium waves, which play crucial roles in the spatiotemporal integration of information within and across cells. Although there have been several demonstrations of regulatory roles of glial cells and dendritic structures in achieving common physiological goals such as information propagation and adaptability through plasticity, studies assessing physiological interactions between these two active structures have been few and far. This lacuna is especially striking given the strong connectivity that is known to exist between these two structures through several complex and tightly intercoupled mechanisms that also recruit their respective ER structures. In this review, we present brief overviews of the parallel literatures on active dendrites and active glial physiology and make a strong case for future studies to directly assess the strong interactions between these two structures in regulating physiology and pathophysiology of the brain.

## Introduction

The role of glial cells and neuronal dendrites, especially involving the multifarious interactions among them, in active information processing in the central nervous system (CNS) has not been fully understood. Glial cells and neuronal dendrites share many commonalities ranging from the integral membrane components (receptors and ion channels) to the presence of endoplasmic reticulum (ER) as a calcium store and the elaborate molecular machinery that sustains active propagation of calcium signals across these structures. Historically, both glial cells and dendritic structures were thought to be passive structures that are not actively involved in information processing. Although decades of research have clearly demonstrated the active nature of glial and dendritic structures and have shown their critical roles in information processing, the potential of how their interactions could contribute to brain functions has not been fully explored. In this review, we discuss various aspects of active physiology and active calcium signal propagation in neuronal dendrites and glia. We present the similarities and differences between glial and dendritic structures, cataloging the impact of interactions between neurons and glia in achieving convergent physiological goals. Importantly, we emphasize the need to systematically study direct interactions between active dendritic and active glial signaling and contend that such interactions and plasticity therein are vital components in encoding, storage, processing, and propagation of information in the CNS.

In the sections below, we first individually introduce calcium stores, glial cells, and active dendrites, also briefly introducing the active nature of signaling through the presence of voltage- and/or calcium-dependent channels and receptors in the plasma membrane and the ER membrane of glial and dendritic structures. Next, we categorize different types of interactions spanning the ER and the plasma membranes of dendrites and glia, outlining the importance of each type of interaction to several aspects of brain physiology. Finally, we present potential future directions for where research on intracellular and intercellular interactions spanning these active structures could be directed towards, also emphasizing the need to systematically assess activity-dependent plasticity in such interactions.

## The Components: Active Glia, Active Dendrites, and Their Endoplasmic Reticulum

Calcium stores in the ER are essential components for calcium signaling across various cell types in the eukaryotes [1–3]. Release of calcium from these stores, through inositol trisphosphate receptors (InsP_3_Rs) and ryanodine receptors (RyRs) expressed on the ER membrane, has been shown to regulate a myriad of physiological and pathophysiological processes in neurons and glia [1, 2, 4–12]. Neuronal ER calcium release plays crucial roles in mediating short- and long-term plasticity and in sustaining actively propagated waves of calcium within and across cells [10, 13–20]. Furthermore, release of calcium from the ER store forms the principal source of cytosolic calcium elevations in the glial cells. Such release of store calcium through InsP_3_Rs in astrocytes enables them to respond to local network cues also through calcium waves. These waves can either be localized within a single cell or travel as intercellular waves propagating into multiple astrocytes that comprises the astrocytic syncytium. Elevations in the cytosolic calcium concentration lead to the release of neuroactive substances from the glial cells which can bind to and activate neuronal receptors, a process termed as gliotransmission [21–29]. These observations about active glial signaling have significantly furthered our understanding of these cells, beyond earlier assumptions that these cells are passive and behave merely as the “glue” that structurally binds neural tissue (the word glia is derived from the Greek word for glue). Together, ER stores in these cell types serve as critical substrates for the integration and transfer of information through the network of neurons and glia across the CNS [8, 11, 12, 22, 24, 27, 30–36].

The functional roles of neuronal dendrites, the elaborate and morphologically complex structures that emanate from the somata, have intrigued neuroscientists for over a century. Classically, neuronal dendrites were also assumed to be passive structures acting as the “receptive apparatus” that funnel the synaptic potentials towards the soma [37–43]. However, the advancements of electrophysiological and imaging techniques have made these fine caliber structures tractable, yielding experimental observations where specific physiologically relevant signals can be directly recorded from these dendritic structures [44–53]. These technological advancements have led to an explosion of information about neuronal dendrites and it is now established that the dendritic plasma membranes express a plethora of voltage-gated ion channels (VGICs). Remarkably, several of these VGICs are expressed heavily in the dendrites with higher densities than at the soma [43, 54–59].

Voltage-gated ion channels in dendritic structures mediate active backpropagation of action potentials [45, 54, 60] and bestow upon dendrites the ability to initiate local dendritic spikes that act as dendritic outputs [61–68]. In addition, these active components in dendritic structures are critical regulators of location-dependent feature selectivity, spike phase coherence, signal integration, and coincidence detection in neurons [51, 54, 58, 66, 69–88]. Importantly, as the structural substrate for most synaptic receptors, channels, and other transmembrane proteins, dendrites are also critically involved in the plasticity of all these components which contribute to the adaptability of neuronal structures to afferent inputs [38, 43, 70, 89–97]. The discovery of plastic active dendrites has resulted in a paradigm shift in our understanding about neuronal information processing, whereby it is now clear that neuronal dendrites play a dominant role in signal integration, neural computation, plasticity, and associated adaptibility in neuronal structures.

Contiguous to the plasma membranes of neurons (extending to dendrites, spines, axons, and boutons) and astrocytes (extending across its processes) runs the ER that forms a continuous membranous network throughout the cytoplasm [1, 10, 98, 99]. The cellular rules governing the resting concentrations of calcium in the cytosol and the ER lumen are in stark contrast to each other. Whereas the resting levels of cytosolic calcium are in the nanomolar range, it is in the high micromolar to low millimolar ranges within the ER lumen [1, 10, 11, 100, 101]. Thus, the ER calcium can be released into the cytosol by activation of either RyRs or InsP_3_Rs which have calcium and InsP_3_ as their endogenous ligands, respectively. There are three isoforms of InsP_3_Rs, with the InsP_3_R1 acting as the principal neuronal subtype and the InsP_3_R2 primarily expressed in the astrocytes [102–106]. Upon appropriate stimulation, these receptors open and release calcium into the cytosol which can have varying spatiotemporal dynamics depending upon the strength of the stimulation.

A unique feature of both the InsP_3_Rs and the RyRs is their bell-shaped dependence on cytosolic calcium levels, with lower cytosolic calcium concentration ([Ca^2+^]_c_) acting as a coactivator and higher [Ca^2+^]_c_ acting as an inhibitor for both of these receptor classes. Thus, at moderate increase in [Ca^2+^]_c_, binding of calcium to InsP_3_Rs, along with InsP_3_, amplifies [Ca^2+^]_c_ increase by enhancing the flux of calcium through the InsP_3_Rs whereas higher [Ca^2+^]_c_ results in inhibition of these channels even in the presence of InsP_3_ [105, 107–110]_._ This dependence of the ER calcium release channels on [Ca^2+^]_c_ also results in varied spatiotemporal characteristics of the ER calcium signaling. For instance, consequent to a relatively weak stimulus and localized mobilization of InsP_3_ within the cytosol, a small number of InsP_3_Rs are activated leading to localized calcium elevation constituting a calcium microdomain—also known as a calcium spark [3]. In contrast, a strong stimulus can lead to a widespread mobilization of InsP_3_ which thus recruits a higher number of InsP_3_Rs on the ER membrane. The calcium dependence of InsP_3_ receptors is dependent on several factors, including the specific subtype of InsP_3_R and their interactions with other signaling components such as cytochrome C [105, 107, 111–113].

Calcium released through these receptors can further diffuse to the nearby receptors at high enough concentrations to synergistically increase the flux of calcium which results in regenerative release of calcium from the ER stores—a mechanism referred to as calcium-induced calcium release (CICR). Through the recruitment of such processes, large amplitude regenerative release of calcium can actively propagate as calcium waves over long distances within a cell. This acts to synchronize and integrate signal processing across various neuronal compartments and is an essential element of biochemical signal processing. The calcium waves can also cross over to the neighboring cells which are connected through gap junctions and by the process of CICR constitute intercellular waves that are prevalent across the glial syncytium [2–5, 10, 16, 19, 107, 114–121].

As the scope of this review is on the interactions between active glia and active dendrites, including interactions with the ER membrane within both structures, we refer to reviews on active dendrites [38, 39, 42, 43, 81, 93, 122], gliotransmission [8, 21, 22, 24, 26–28, 123–125], and ER signaling in neurons and glia [1–4, 6, 7, 10–12, 19] for further details on each of these individual components. In what follows, we assess interactions across these components and associated plasticity mechanisms.

## Trees and Stores: Active Dendrites and ER Membrane

Several neuronal subtypes across the brain are endowed with extensive dendritic arborization. In pyramidal neurons of the cortex and the hippocampus, the neuronal architecture is such that several thin caliber oblique dendrites form branches of a main apical dendritic trunk, with several basal dendrites emanating from the cell body [126–129]. On their plasma membrane are present several VGICs with varying biophysical properties and subcellular distributions [38, 43, 56, 57, 93, 130]. Parallel to the neuronal plasma membrane is the ER membrane that forms a continuous network throughout the neuronal morphology. Upon activation of specific metabotropic receptors or elevation of cytosolic calcium through other sources, the InsP_3_Rs and RyRs present on the ER membrane respond by releasing calcium into the cytosol, which can exhibit diverse dynamics depending upon the strength of stimulation [3, 16, 19, 47, 48, 114–116, 120, 131]. Such a structure constitutes two parallel active membranes (the ER and the plasma membrane), which are endowed with channels, receptors, pumps, and other transmembrane components, that participate in active propagation and integration of information across the neuronal structure.

In addition to the structural contiguity of the ER and the plasma membranes in neuronal structures, there are profound functional similarities with reference to signal propagation across these two membranes [132]. Whereas the neuronal plasma membrane with its channels, receptors, and pumps mediates the electrical signal propagation, the ER membrane and its receptors participate in the calcium signal propagation along the dendritic length. A main source of excitatory electrical potentials in the dendrites is sodium entry through ionotropic synaptic receptors which leads to membrane depolarization that propagates towards the soma. The spatiotemporal spread of such signals is determined by the extent of dendritic filtering based on the passive and active properties of the dendritic compartments. Under a purely passive propagation, the magnitude of decay in such synaptic potentials is determined by the electrotonic length constant (*λ*_E_) of the dendrites [133]. *λ* denotes the distance at which a propagating signal attenuates to 37% of its initial amplitude. For a neurite with the same set of passive parameters, a time-varying signal (such as excitatory post synaptic potentials, EPSPs, and action potentials, AP) undergoes heavier attenuation compared to a steady state signal. Thus, the length constant for the voltage signal obtained in response to a direct current injection (*λ*_DC_) is greater than the length constant with reference to a time-varying signal (*λ*_AC_). Furthermore, the faster the kinetics of a time-varying signal, the higher is its attenuation as it propagates passively, implying that an action potential would attenuate much more than an EPSP for the same distance on the same cable [133].

Although this is the scenario under passive propagation, under physiologically realistic conditions, a synaptic potential is subjected to modifications by both passive as well as active properties of the dendrites. This is effectuated through ornate spatiokinetic interactions between the propagating potential and various VGICs [38, 54, 73, 91, 93, 134–140]. Generation of AP that constitutes an active regenerative signal propagation involves a positive feedback loop where a small amount of depolarization leads to further opening of the fast sodium (NaF) channels thereby leading to more depolarization of the membrane, ultimately giving rise to a fast deflection in membrane voltage. Following the voltage-dependent activation, conformational changes in NaF channels result in their inactivation such that the inactivation is indirectly voltage-dependent. Subsequent voltage-dependent activation of high threshold delayed rectifier K^+^ channels completes the repolarization of the membrane. Thus, the AP wave propagates through voltage depolarization of the membrane [134, 141–145].

The ER membrane can also participate in the passive and active calcium-based signal propagation. Analogous to the attenuating passive propagation of electrical potentials, a relatively small flux of calcium through the ER receptors or voltage-gated calcium channels leads to passive diffusion of calcium to nearby locations. The extent of such diffusion is determined by the diffusion coefficient of calcium ion in the cytosol, binding to calcium buffers and several “off” mechanisms that result in the extrusion of calcium from the cytosol [3, 120, 146]. The attenuation in the calcium signal can be quantified by a space constant for calcium decay within the cytosol, denoted by *λ*_Ca_. Notably, elaborate cellular calcium handling mechanisms lead to stringent control of cytosolic calcium elevations, thereby causing *λ*_Ca_ to be smaller than *λ*_E_ [132, 147]. This reflects compartmentalization of the downstream signaling pathways that the elevated calcium elicits and is crucial for establishing micro- and nanodomains of calcium signaling [3, 120, 146, 148, 149]. During active calcium signal propagation, CICR-dependent amplification of the ER calcium release constitutes a positive feedback loop resulting in large elevations in the cytosolic calcium. This steep rise in the cytosolic calcium then acts as an inhibitor for the ER receptors (due to the bell-shaped dependence of the InsP_3_Rs and RyRs on the cytosolic calcium), thereby shutting the flux of calcium from the ER. Thus, the active propagation of calcium signal encompasses regenerative release of calcium from the ER stores that propagates in the form of calcium waves [2, 3, 19, 105, 107, 146].

Although there are significant qualitative equivalences in active and passive signal propagation across the active dendritic plasma membrane and the active ER membrane, there are several quantitative differences in terms of the spatial and temporal spread of these signals and the mechanisms that govern such spread. Specifically, the calcium signals are typically slower than their electrical counterparts, although their spatial spread is much constricted compared to electrical signal spread.

## Waves in Trees: Parameters Governing Active Dendritic Calcium Wave Propagation

Calcium waves have been observed in various neuronal subtypes [3, 16, 19, 48, 120, 150, 151]. They constitute large amplitude elevations in the cytosolic calcium which can rise up to a few micromolars and last for about 1–2 s. Calcium waves can be elicited by physiologically relevant synaptic stimulations as well as pharmacological agents that lead to the mobilization of cytosolic InsP_3_. Specifically, synchronous synaptic stimulation results in a delayed increase in the cytosolic calcium as opposed to the fast and relatively small influx of calcium through the opening of synaptic receptors and VGCCs during the stimulation. The resultant wave propagates regeneratively to a distance of several tens of microns by recruiting CICR from the nearby clusters of InsP_3_Rs on the ER membrane. Furthermore, when synaptic stimulation is paired with a short train of somatically induced APs, the depolarization-induced calcium influx leads to a synergistic increase in the calcium release from the ER stores. This enhances secondary elevation in the cytosolic calcium which expedites initiation and propagation of calcium waves in the apical dendritic shaft [16, 19, 115, 116, 151–154]. Mechanistically, the initial trigger for the wave initiation is provided by the activation of group I metabotropic glutamate receptors (mGluRs) and consequent mobilization of cytosolic InsP_3_ during synaptic stimulation [3, 4, 16, 19, 116, 155].

Synaptically activated calcium waves always initiate at the branch point of the oblique dendrite on the apical dendritic trunk closest to the stimulating electrode [115]. Furthermore, even when the synaptic stimulation is paired with somatic APs, these waves are not able to invade the soma. The low surface area to volume ratio at the soma effectively acts as a sink thereby diluting the concentrations of InsP_3_ and calcium that diffuse into the soma, together disrupting the regenerative flux of calcium from the ER stores [19, 115, 153, 156]. Consistently, when the calcium waves are elicited by direct infusion of InsP_3_ into the soma (through a patch pipette) or by bath application of mGluR1 agonist 1-aminocyclopentane-*trans*-1,3-dicarboxylic acid (t-ACPD), they can invade the soma [116, 152, 153]. These experiments establish that the functional InsP_3_Rs are indeed present at the soma of these neurons. Concordantly, immunohistochemical and electron microscopic studies have also established the presence of InsP_3_Rs in neuronal soma, dendrites, and axons. Interestingly, in the CA1 pyramidal neurons the highest density of InsP_3_Rs is present in the somatic layer which monotonically decreases towards the distal apical dendrites [102, 104, 106, 157].

**Fig. 1:**
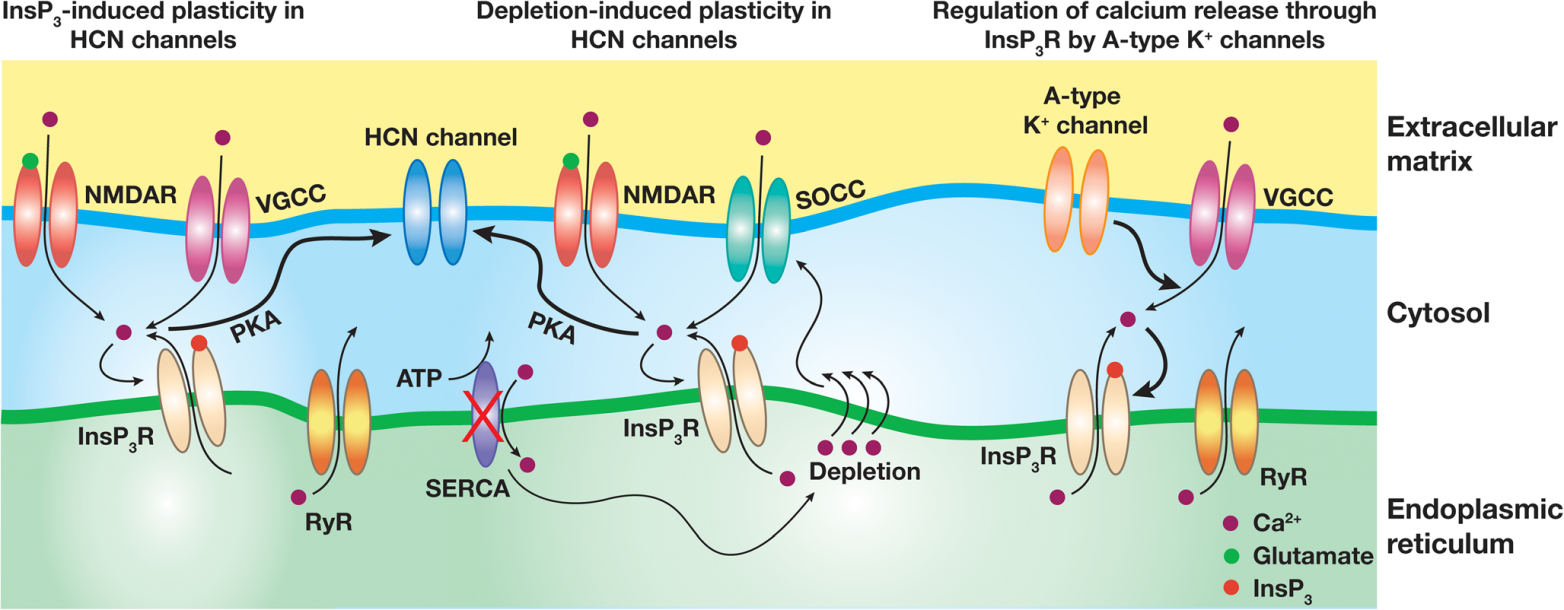
A diagrammatic representation of the interaction between various VGICs and receptors present on the plasma membrane with the calcium handling mechanisms on the ER membrane. Thick arrows depict the functional interactions between the connecting molecules. Thin arrows point to the flux of calcium ions through various channels and receptors. SERCA, sarcoplasmic endoplasmic reticulum ATP-ase pump; SOCC, store-operated calcium channels; PKA, protein kinase A; RyR, ryanodine receptor. Based on data from [17, 156, 158]

Although several structural and functional similarities exist between the ER and the plasma membranes, a largely unaddressed question is how the dendritic plasma membrane interacts with the ER membrane and its components to shape neuronal physiology (Fig. 1)? In the past decade, various kinds of interactions between the ER and the plasma membranes have been investigated that have opened new avenues towards our understanding of the ER-plasma membrane interactions. One of the most prominent form of such interactions is the influx of calcium through store-operated calcium channels that are formed in response to the depletion of ER calcium stores [9, 159–161]. Depletion of the ER calcium stores leads to conformational changes in the stromal intercalation molecules (STIM) protein present on the ER membrane. This in turn induces formation of ER membrane and plasma membrane junctions where the Orai proteins cluster on the plasma membrane and interact with the STIM proteins leading to the formation of active calcium release activated calcium (CRAC) channels. This is one of the principal mechanisms to fill the depleted calcium stores in electrically non-excitable cells [159–162]. Interestingly, in the electrically excitable cells STIM can also interact with the Cav1.2, which constitutes the *L*-type voltage-gated calcium channels (VGCCs), to inhibit the calcium entry through these channels [163]. Thus, plasma membrane calcium sources and ER calcium sources can interact with each other in a mutually interdependent manner to enhance or reduce intracellular calcium levels. Furthermore the ER calcium release can also regulate electrical excitability of the cortical and hippocampal neurons through the calcium-dependent inhibition (CDI) of VGCCs and through the activation of calcium-gated small conductance potassium (SK) channels [153, 164–167].

Given such tight interactions between the ER and the plasma membranes, how do various types of VGICs interact with the ER membrane? For instance, apart from VGCCs, there are other VGICs which critically regulate the excitability and hence the influx of calcium into the neuronal cytosol. Specifically, in the light of the bell-shaped dependence of InsP_3_Rs activation on the cytosolic calcium, how would the presence (or absence) of various VGICs translate into the regulation of store calcium release through InsP_3_Rs? In addressing this, the impact of a restorative conductance mediated by *A*-type K^+^ (KA) channels on the store calcium release was quantitatively investigated [156]. Specifically, calcium waves were elicited in a morphologically and biophysically realistic model of CA1 pyramidal neurons with biochemically constrained calcium handling components that were implemented as a well-mixed reaction-diffusion system. Within this framework, the flux of calcium through the InsP_3_Rs followed a bell-shaped dependence on the density of KA channels, directly consequent to the regulation of InsP_3_Rs by cytosolic calcium. To elaborate, calcium waves in these models were initiated through an experimentally validated protocol where, increased level of cytosolic InsP_3_ (corresponding to the bath application of Group1 mGluR agonist) was paired with a short train of APs [16]. When the initial KA conductance was low, the influx of calcium through the VGCCs, during the wave initiation, was high enough to inhibit further release of calcium through the InsP_3_Rs. As the KA conductance was increased in these neurons, the flux of calcium through the VGCCs dropped to a level where it was optimal to act as the coactivator for the InsP_3_Rs leading to an increase in the flux of calcium through these receptors. Further increase in the KA conductance led to reduction in this “activator” level of calcium in the cytosol and hence reducing the synergistic action of cytosolic calcium on the InsP_3_Rs. This resulted in a decrease in the flux of calcium through the InsP_3_Rs, thus giving rise to a bell-shaped dependence of InsP_3_R opening on the KA conductance.

Additionally, analogous to the regulation of EPSPs and backpropagating action potentials by KA channels, the presence of KA conductance also regulated the kinetics of calcium waves in these neuronal models, and manifested as increased latency to peak and enhanced temporal width of the calcium waves [54, 156]. Notably, this dependence of InsP_3_Rs on KA conductance unmasks a novel form of interaction between the ER and the plasma membranes where dendritic excitability can potentially regulate the biochemical signal integration by steering the spatiotemporal spread of calcium. Specifically, as these results demonstrate that the spread of calcium can be critically regulated by interactions between membrane proteins on the plasma membrane and the ER membrane, the spatiotemporal spread of downstream signaling components (that are reliant on calcium as the second messenger) could also be altered by such interactions [149, 168–170]. Therefore, we postulate the presence of voltage-gated channels and their interactions with the ER membrane could steer the activation and spread of biochemical signaling through the relative localization of ion channels on either membrane and that of biochemical signaling components. Interestingly, similar to the results of in vitro slice experiments [115], calcium waves always originated at the branch points in this modeling study also. As several parameters can be independently and precisely controlled in the modeling study, this opened avenues to investigate various morphological, biophysical, and calcium handling mechanisms that could contribute to branch point initiation of calcium waves.

A systematic investigation of various neuronal parameters revealed neuronal morphology, through control of the reaction-diffusion process by regulating the surface area to volume ratio (SVR), to be a critical regulator of wave initiation and propagation in these morphologically elaborate neurons. This modeling study also revealed that changes in InsP_3_R density regulate the wave amplitude without altering the location of its initiation. To elaborate, high SVR in thin caliber dendrites translates into a large build-up of calcium in these compartments in response to the AP-induced opening of VGCCs. Thus, in the absence of any restorative conductance this initial calcium concentration is high enough to act as an inhibitor for the InsP_3_Rs in oblique dendrites. However, in the presence of KA channels which express in high densities in the oblique dendrites [54, 55, 63, 171, 172], the initial calcium influx is reduced to be in the permissive range that can synergistically activate InsP_3_Rs to initiate a calcium wave. The calcium from these compartments can then actively propagate to the main apical dendritic trunk, further amplifying the calcium release at the branch point through CICR. Together, this manifests in the branch point initiation of calcium waves in these neurons, and the expression of KA channels make the stores and InsP_3_R there to be relevant for information processing in obliques (without these channels, the InsP_3_R would just be inhibited by the initial large influx of calcium). These observations suggest that KA channels can regulate both the spatial propagation as well as the temporal aspects of calcium waves, by regulating the dendritic excitability, under various physiological and pathophysiological conditions. Furthermore, the expression of KA channels together with the localized plasticity that these channels can undergo [94, 95] would allow them to regulate the spread of calcium microdomains towards specific subregions in the dendrites.

Apart from KA channels, there are other conductances expressed on the active dendritic membrane such as the hyperpolarization-activated cation non-specific (HCN) channels and the *T*-type calcium channels. In addition to their expression at the soma [58, 59, 173], these channels are also present in high densities in the neuronal dendrites and regulate the cytosolic calcium concentrations by either directly mediating the influx of calcium (*T*-type calcium) or indirectly by controlling the dendritic excitability (HCN) [3, 59, 71, 120, 174, 175]. Future experimental and computational lines of investigation are required to delineate the roles of these and other channels in regulating the release of calcium from the ER store and understand the impact of such interactions on the neuronal physiology.

## Dynamic Trees and Stores: Plasticity and Cross Regulation Across the Two Active Membranes

From the description above, it is clear that plasma membrane channels and receptors can regulate ER calcium release through the dependence of ER calcium channels on cytosolic calcium levels and through mechanisms of capacitative calcium entry through store-operated calcium channels (SOCs). Alternately, one can ask whether the release of calcium through the receptors on ER membrane results in reciprocal regulation of receptors and ion channels located on the plasma membrane? Indeed, the release of calcium from the ER stores critically regulates the extent and polarity of several forms of neuronal plasticity through regulation of synaptic receptors [1, 10, 13, 14, 20, 95, 176–178]. Notably, activation of InsP_3_Rs is necessary for the induction of certain forms of heterosynaptic plasticity, thus highlighting the role of these receptors in regulating neuronal physiology through long-range propagation of calcium signals [20, 177].

ER calcium release can also induce plasticity of voltage-gated ion channels present on the plasma membrane under various physiological and pathophysiological conditions. As a specific instance of such plasticity, consider disruptions in the calcium homeostasis in the ER, which has been observed as part of several pathological states and has been associated with multiple neurological disorders [1, 179–181]. Experimental imitation of such ER stress response can be achieved by blocking sarcoplasmic endoplasmic calcium ATP-ase (SERCA) pumps that leads to the depletion of ER calcium stores. Such depletion of ER stores results in a decrease in the neuronal excitability and an increase in the optimal response frequency of CA1 pyramidal neurons [17]. This plasticity of the intrinsic response dynamics (IRD) is mediated by an increase in the functional density of HCN channels and associated changes in neuronal biophysical properties [17, 182, 183]. Notably, pharmacological blockade of either the flux of calcium through InsP_3_Rs or SOC channels abolished this form of plasticity suggesting the necessity of InsP_3_R activation for the induction of such plasticity [17]. The role of SOC channels in inducing plasticity of intrinsic neuronal properties is not confined to the hippocampus, but has been shown in other brain regions as well. For instance, the activation of Orai1 has been recently shown to enhance neuronal excitability and to reduce the current through KA channels, mediated by the PKC-ERK signaling cascade in dorsal horn neurons [184].

The store depletion-induced plasticity in hippocampal HCN channels and intrinsic excitability is postulated to serve as a neuroprotective mechanism where reduced excitability can protect the neurons from excitotoxicity in the face of aberrantly high network activity [17, 185]. More recently, it was demonstrated that depletion of ER stores in vivo through infusion of a SERCA pump inhibitor into the dorsal CA1 region induced anxiogenic-like behaviors, apart from enhancing the current through HCN channels. This was found to be similar to the enhancement of perisomatic HCN1 protein expression and physiological correlates pointing to enhanced HCN channel function, which were observed with chronic unpredictable stress [186]. Together with previous studies on the antidepressant roles of knocking down HCN1 channels from dorsal hippocampus [187], these results point to a critical role for ER calcium stores and their interactions with plasma membrane voltage-gated channels in depressive disorders [186–188].

In another study, designed to assess the sufficiency of InsP_3_ receptor activation on plasticity of intrinsic neuronal properties, direct infusion of InsP_3_ into CA1 pyramidal cells resulted in a similar form of intrinsic plasticity as was observed with ER store depletion [158]. Thus, these sets of experiments established that the activation of InsP_3_Rs is both necessary as well as sufficient for the induction of plasticity of HCN channels expressed on these neurons [17, 158, 182, 186]. Strikingly, the InsP_3_-induced plasticity is graded, whereby higher activation of InsP_3_Rs resulted in higher amount of plasticity in the IRD measurements. This implies that under physiological conditions, the ER stores induced plasticity of intrinsic properties can express over a wide dynamic range, in a manner that is quantitatively dependent on metabotropic synaptic activity and consequent graded mobilization of cytosolic InsP_3_. This implies that the magnitude of plasticity in the IRD can be tuned to optimize neuronal response depending on the state of the network activity and represents a cellular mechanism that enables a neuron to maintain its dynamic range of activity by fine tuning its gain over a varied range of network activity (by adjustment of plasma membrane ion channels). In addition to these results pertaining to HCN channels, in the hippocampal cultured neurons, ER calcium release through RyRs has recently been shown to be necessary to effectuate downregulation of *A*-type potassium channels [189].

Together, it is clear that different signaling cascades differentially recruiting the activation of distinct calcium release channels on the ER membrane can regulate the dendritic excitability by acting on disparate plasma membrane VGICs. It should however be noted that these recent advances reporting such ER-induced plasticity in plasma membrane ion channels, thereby altering the gain and intrinsic response dynamics of neurons, constitute only the tip of the iceberg. There is a large repertoire of receptors and channels present on the plasma membrane which can potentially be regulated by the ER calcium release, locally (the perisomatic plasticity in HCN channels is an example for local regulation) or globally (especially given the spread of the ER across the neuron) with different patterns of release (e.g., tonic vs. phasic with different frequencies and patterns). Given the strong links between ER stores to neuronal physiology and pathophysiology and given the several roles of voltage-gated ion channels in neurophysiology and associated channelopathies in neurological disorders, it is clear that a systematic analysis is required to uncover ER-induced intrinsic plasticity across different cell types to assess the impact of such regulation on neuronal information processing and encoding.

## Stores, Waves, and Glue: ER Stores and Calcium Waves in the Glial Syncytium

Glial cells are cell types that are electrically non-excitable and derive their name from the Greek word for glue as they were first thought to constitute the binding material for the neuron in the brain. Later with the advancement of cellular staining techniques, it became clear that the neuroglia constitute a distinct class of brain cells. Ever since, our understanding about the function of these cells has grown tremendously and it is now evident that they critically participate in regulating information propagation and processing along with metabolism in the brain [21, 190–197].

Astrocytes are a subclass of glia cells which are morphologically complex with cell bodies that appear star-shaped. Due to lack of electrogenic sodium channels [198–200], they were long considered to play a supportive role in the central nervous system where they provide metabolic support and optimize vascular supply to different brain regions. However, in the latter part of the twentieth century, with the advent of calcium imaging techniques, it became clear that astrocytes are calcium excitable, whereby they respond to neuronal activity by increase in their cytosolic calcium levels [8, 21, 29, 117, 118, 201]. Furthermore, calcium imaging along with genetic manipulations of astrocytes have presented compelling lines of evidence that the astrocytes are integral components of information processing in the brain that communicate among themselves (predominantly through gap junctions) as well as with neurons [21, 24, 32, 33, 35, 191, 193, 194, 196]. More recent studies have also uncovered critical roles for astrocytes in the regulation of animal behaviors such as sleep, breathing, mastication, and in the control of circadian rhythm [190, 196, 202–206].

Astrocytes respond to varied sensory stimuli through changes in their calcium signals in vivo [207–209]. Similar calcium signals are also observed upon stimulation of axonal afferents suggesting that astrocytes respond to neuronal network activity. Specifically, glial cells respond to the release of neurotransmitters (through activation of metabotropic receptors expressed on their plasma membrane), leading to mobilization of cytosolic InsP_3_ and subsequent release of calcium from the ER stores. This can induce intracellular calcium waves, which can propagate to neighboring astrocytes through gap junctions to constitute intercellular calcium waves that can travel through the astrocytic syncytium [11, 117, 118, 190, 210, 211]. Astrocytic calcium elevation translates into the release of several neuroactive chemicals from astrocytes—termed gliotransmission—that regulate a myriad of neurophysiological processes including synaptogenesis, synaptic transmission and plasticity, neuronal excitability, action potential propagation, and modulation of neuronal synchrony and behavior [22, 29–31, 34, 203, 212–215].

Individual astrocytes are morphologically elaborate with very fine protoplasmic processes that make close contacts with several neurons and tens of thousands of synapses [8, 123, 196, 216–218]. At synaptic junctions, individual astrocytic processes respond to the neurotransmitters that diffuse around the synaptic cleft. This response is achieved through the activation of high-affinity receptors located on the astrocytic processes, which in turn elicit cytosolic calcium elevation and consequent release of gliotransmitters from the astrocytes. The gliotransmitters act upon various pre- and/or post-synaptic neuronal receptors to modulate synaptic activity, thus making the synaptic information transfer tripartite, where astrocytes are crucial regulators of information transmission and processing [12, 21, 22, 24, 26, 28, 34, 191, 212, 219–221].

## Glue, Stores, and Trees: Active Neuronal Dendrites and Gliotransmission

One of the most direct impacts of gliotransmission on neuronal excitability is the emergence of slow inward currents (SIC) in neighboring neurons. Specifically, glutamate released by the astrocytes can act on the extrasynaptic *N*-methyl-d-aspartate receptors (NMDARs) to elicit SICs in the proximal neurons [22, 31, 211, 213, 222–225]. Notably, the frequency of SICs is dependent on the extent of astrocytic activation. For instance, synchronous stimulation of Schaffer collaterals in hippocampal slices and consequent excitation of group 1 mGluRs on the astrocytes leads to an increase in the frequency of SICs in CA1 pyramidal neurons [211]. Furthermore, apart from releasing glutamate, astrocytes are capable of releasing various other gliotransmitters that play important roles in the modulation of synaptic transmission and plasticity in various brain regions [21, 22, 206, 226–228].

Although the phenomenon of gliotransmission and its impact on neuronal physiology has been reported to be widespread across different brain regions, the mechanisms behind gliotransmitter release have been debated. It has now emerged that several mechanisms may contribute to gliotransmitter release. For instance, although there is direct evidence for the vesicular release of glutamate in a calcium-dependent manner, non-exocytotic release of glutamate through gap junction hemichannels, swelling activated anion channels, and reverse operation of glutamate transporters have also been reported under different experimental conditions [8, 12, 32, 124, 190, 222, 229–237]. Additionally, different neurotransmitters can be released through similar molecular machinery. For instance, in the hippocampal astrocytes, bestrophin-1 (BEST-1) receptors mediate direct release of glutamate from the astrocytes, whereas in the cerebellar glia, these channels mediate the release of GABA [238, 239].

Adding further complexity to the calcium-mediated gliotransmitter release is the fact that there are diverse mechanisms of cytosolic calcium elevation in astrocytes that may be activated by divergent upstream signaling mechanisms through activation of different plasma membrane bound GPCRs. For example, in hippocampal astrocytes of type 2 InsP_3_R knockout mice, not all spontaneous calcium signals are abolished suggesting that other sources of calcium (mediated by other receptors and channels) can play a role in the emergence of such signals [123, 218]. Additionally, activation of group-1 mGluRs as well as the protease activated receptor 1 (PAR-1) or P_2Y1_-purinoreceptors can lead to similar calcium excitability in astrocytes. Whereas pharmacological activation of group-1 mGluR and PAR-1 leads to the gliotransmission-induced SICs in the proximal neurons, the activation of P_2Y1_-purinoreceptors does not [223].

Astrocytic calcium signaling spans a wide range of spatiotemporal characteristics. Notably, calcium microdomains localized to the fine astrocytic processes that lie in close proximity of the neuronal dendrite can potentially induce localized and heterogeneous neuron-astrocyte interactions [12, 33, 123, 125, 217, 240, 241]. Specifically, three-dimensional imaging from astrocytic soma and their fine processes has shown calcium activity in these structures to be highly heterogeneous, with different parts of the astrocyte showing significant asynchronous calcium activity [241]. These observations suggest that the dendritic structures that are apposed to different parts of an astrocyte might receive differential localized activation. Yet, much of our understanding about the gliotransmission is based on neuronal somatic recordings, thereby limiting our understanding about the spatial impact of gliotransmission on electrotonically non-compact neuronal structures. Because of the profound impact that gliotransmission has on the neuronal physiology and the fact that nearly 80% of the total synaptic connections are present on dendrites, it is important to assess the effect of localized gliotransmission on neuronal dendrites. Furthermore, complex spatiokinetic interactions among the VGICs expressed on the neuronal dendrites critically regulate signal integration and processing underlying neuronal physiology [39, 42, 43, 77, 136] and can potentially regulate the impact of gliotransmission too. Therefore, to understand the emergence and spread of gliotransmission-induced neuronal events, it is necessary to record these events directly from the dendrites. This can answer whether their impact is limited to specific neuronal compartments, and hence local, or whether they have widespread impact to serve as global modulators of neuronal physiology.

Direct recordings of the voltage counterpart of SICs as slow excitatory potentials (SEPs) from the dendrites of CA1 pyramidal neurons reveal large amplitude voltage deflections in the distal dendritic region [242]. Specifically, the peak amplitude of SEPs in the distal dendrite is about fourfold higher than those recorded at the soma. Additionally, the rise time of spontaneous SEPs (sSEPs) in the distal dendrites (~ 200 to 250 μm away from the soma) is lower than somatic SEPs. These observations, along with simultaneous somatic and dendritic recording of sSEPs, reveal predominantly dendritic origin of SEPs which are subjected to dendritic filtering as they propagate towards the soma. Notably, the dendritic SEPs could reach amplitudes of tens of millivolts and span hundreds of milliseconds in duration [242]. This is in striking resemblance with the voltage waveform of plateau potentials observed in these neurons under in vivo and in vitro conditions [243–246]. This crucial evidence suggests that gliotransmission can heavily impact dendritic information processing and plasticity by eliciting localized plateau potentials in these neurons.

These direct dendritic recordings of the impact of gliotransmission also revealed that the spatial localization of SEPs is brought about by the active dendritic mechanisms. Specifically, pharmacological blockade of KA and HCN channels—the two prominent VGICs which are heavily expressed on the dendrites of these neurons—uncover two distinct mechanisms for the spatiotemporal localization of SEPs [242]. Blocking KA channels, specifically in the neuron being recorded through intracellular infusion of pharmacological blockers, results in an increase in the amplitude of dendritic but not somatic sSEPs with no significant change in their kinetics and frequency. In contrast, blocking HCN channels, again specifically in the neuron being recorded through intracellular infusion of HCN channel antagonists, does not alter the amplitude of somatic and dendritic sSEPs. However, with blockade of HCN channels there is a significant increase in the rise time, duration, and frequency of dendritic, but not somatic, sSEPs.

Direct dendritic recordings also reveal the SEPs impinge upon the neuronal arbor at much higher frequency than previously estimated by somatic recordings, as several of them were significantly attenuated before they reach the soma due to compartmentalization by active dendritic mechanisms. As mentioned above, the blockade of HCN channels, one such dendritic mechanism involved in active compartmentalization, reveals the higher frequency of SEPs impinging on the neuronal arbor. Mechanistically, blockade of HCN channels increases the intercompartmental coupling [70, 77, 78, 247], thereby resulting in more effective propagation of SEPs towards the dendritic location being recorded [242]. The higher expression of HCN channels in the dendrites therefore translates into a higher impact of their regulation of dendritic SEPs (in comparison to somatic SEPs), together manifesting as an increase in the frequency of dendritic sSEPs when HCN channels are blocked [242].

**Fig. 2:**
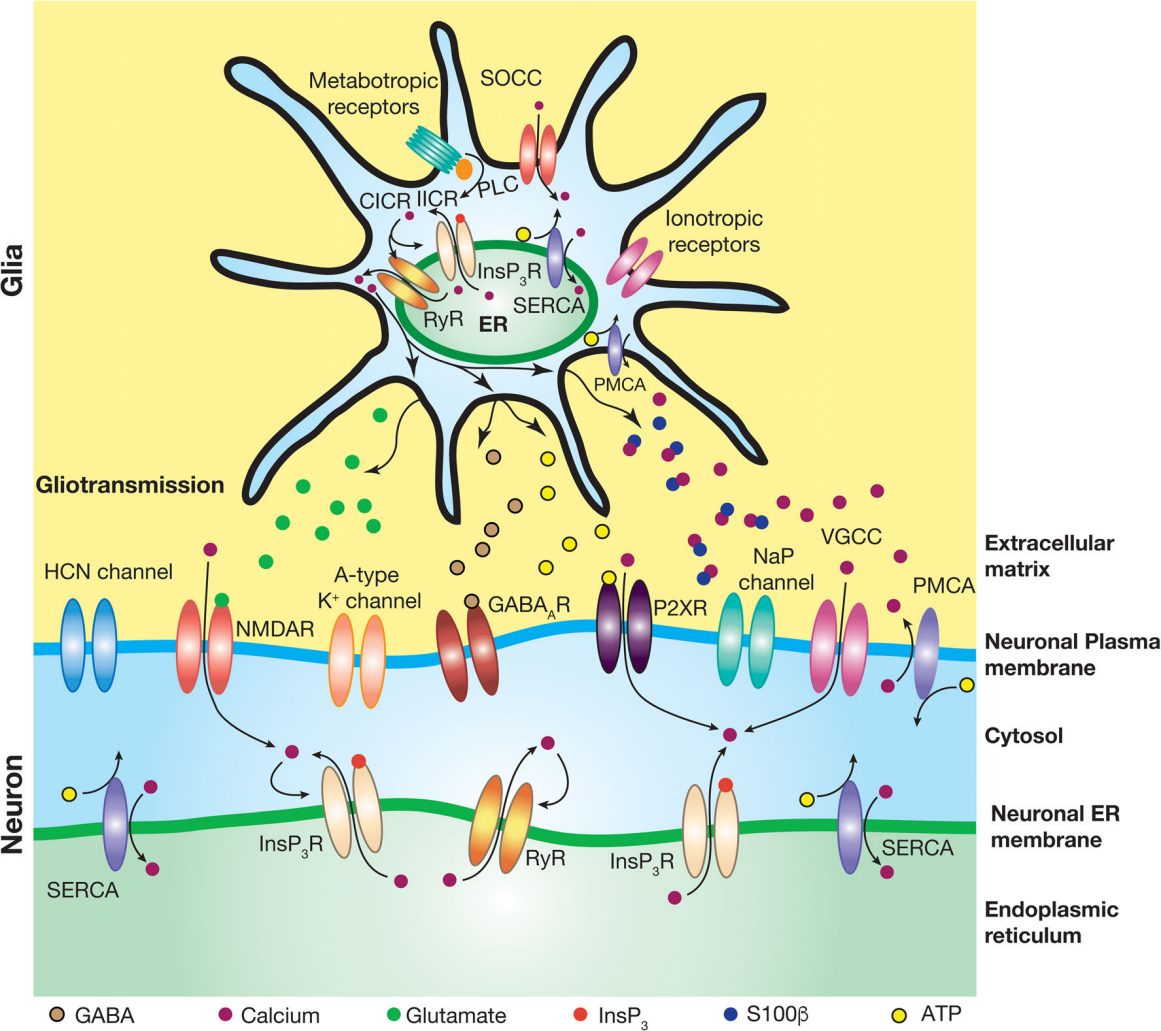
Multifarious interactions between active glial signaling and active dendritic components. The roles of different gliotransmitters and their neuronal receptors have been studied across various systems and different brain regions. Gliotransmission of different transmitter molecules activate associated receptors on the postsynaptic neuronal membranes. The impact of gliotransmission on dendritic membrane is regulated by the presence of voltage-gated channels (e.g., HCN and *A*-type potassium) on the dendritic membrane. Glial release of S100β reduces extracellular free calcium by binding to them, thereby reducing the suppression (by extracellular free calcium) of persistent sodium (NaP) channels on the neuronal membrane. Pumps and transporters present on the glial and dendritic plasma membranes also contribute to the regulation of extracellular ionic concentration and homeostasis. Ionotropic and metabotropic receptors on the glia can be activated by neurotransmission and those on neurons can be activated by gliotransmission, forming another form of interaction between glial and dendritic structures. Store-operated calcium channels have been shown to be present on neuronal and glial plasma membranes. IICR, InsP_3_-induced calcium release; CICR, calcium-induced calcium release; PLC. phospholipase C; SOCC, store-operated calcium channels; PMCA, plasma membrane calcium extrusion pump; NaP channels, persistent Na^+^ channels; GABA_A_R, γ-aminobutyric acid receptor type A; P2XR, purinergic P2X receptor; RyR, ryanodine receptor

From the perspective of implications, the localized nature of large amplitude SEPs which are mediated by extrasynaptic NMDARs would translate into local build-up of [Ca^2+^]_c_ restricted to specific neuronal compartments. Consequently, the ensuing plasticity in the neuronal ion channels and synaptic receptors would also be localized [42, 96, 244–246]. Conversely, spatially restricted plasticity in the VGICs that regulate SEPs [95, 96] would translate into local regulation of the spatiotemporal spread of SEPs by the active dendrites. Taken together, active dendritic mechanisms add an additional layer of complexity to neuron-astrocyte interactions. This presents a scenario where gliotransmission, mediated by the astrocytic ER calcium release, can regulate the receptors and ion channels present on the neuronal plasma membrane (Fig. 2), which in turn could regulate ER calcium release in neurons (Fig. 1).

Yet another line of evidence reflecting the intricacies of the complex neuron-astrocyte interactions is exemplified by the fact that the astrocytic ER calcium release can also be triggered by the activation of astrocytic GABA_B_ receptors upon release of GABA from interneurons. Consequently, astrocytic glutamate release facilitates glutamatergic synaptic transmission through activation of presynaptic mGluRs the hippocampal microcircuit [248]. Thus, multiple mechanisms of functional interactions between the astrocytic ER stores and neuronal receptors and VGICs heavily impact neuronal information processing in the brain [196, 197].

This impact of gliotransmission on behaviorally relevant neuronal computations is further exemplified by the emergence of gliotransmission-induced plateau potentials in CA1 pyramidal neurons [242]. Notably, in vivo patch clamp recordings reveal that during a virtual navigation task, non-place cell neurons can give rise to place cells subsequent to the occurrence of plateau potentials in successive trials [245]. Furthermore, artificial induction of plateau potentials, by depolarizing current waveforms, can convert a non-place cell into a place cell [245, 246], possibly through depolarization-induced calcium influx which can then induce task-dependent neuronal plasticity. Importantly, this behavioral regime where the emergence of place cells is accompanied by impingement of synchronous synaptic inputs and associated plateau potentials is strikingly similar to the conditions of synaptic activation that results in astrocytic calcium excitability and consequent gliotransmission [211]. Thus, taking all these together, we postulate that the gliotransmission-induced plateau potentials recorded in the hippocampal neurons represent one of the cellular mechanisms through which astrocytes could contribute to the emergence of place cells in the hippocampus.

An important form of interaction between astrocytes and ion channels on neuronal plasma membrane is mediated by ions in the extracellular space. Specifically, astrocytes play critical roles in the regulation of ionic homeostasis in the extracellular space, which alter the local reversal potentials of and/or modulate neuronal ion channels, thereby acting as regulators of intrinsic excitability of nearby neurons under various physiological and pathophysiological conditions [125, 249–252]. Recent studies on the trigeminal main sensory nucleus [206, 249, 253] have unveiled an additional role for such interactions (Fig. 2) through glial release of S100β [254, 255]. Increase in extracellular S100β, a calcium binding protein, decreases the concentration of extracellular free calcium ([Ca^2+^]_o_), which in turn reduces the suppression of persistent sodium channels by [Ca^2+^]_o_ [206, 249, 253, 256–259]. The net effect of glial release of S100β is thus a shift in the output mode of the neurons from tonic firing to bursting through augmentation of the persistent sodium current [206, 249, 260, 261]. Recently, persistent sodium channels have been demonstrated to play a critical role in mediating the steep voltage dependence of place encoding in hippocampal neurons [262]. In light of this, the contributions of hippocampal glia-neuron interactions, involving extracellular calcium and persistent sodium channels, to spatial encoding should be assessed more carefully. This is especially critical because [Ca^2+^]_o_ has been demonstrated as a critical regulator of persistent sodium current-dependent burst-firing in hippocampal pyramidal neurons [256].

## Dynamic Trees and Glue: Glia-Mediated Plasticity in Neuronal and Synaptic Properties

Astrocytes critically regulate synaptic transmission and network dynamics through a variety of mechanisms. Functional interactions between astrocytes and neurons sculpt synaptic maturation during the development and set the tone for the basal synaptic transmission in the adult brain [33, 34, 214, 263, 264]. Furthermore, several lines of evidence establish their roles in regulation of short- and long-term synaptic plasticity [35, 196, 214, 265–270].

In spite of a large body of literature exploring roles of glial cells in synaptic plasticity, their roles in the regulation of intrinsic neuronal plasticity has not been investigated. The large amplitude dendritic plateau potentials that are consequent to gliotransmission are mediated by NMDARs [242], thereby resulting in a large calcium influx into the cytosol. From lines of evidence from the active dendritic plasticity literature [38, 43, 70, 89, 90, 94–97, 271, 272], it is clear that the downstream signaling cascades associated with this calcium elevation is unlikely to be specific for synaptic receptors and are expected to induce plasticity of voltage-gated channel properties as well. As a consequence, an important future direction for astrocyte-neuron interactions is whether gliotransmission can induce plasticity of neuronal intrinsic properties through regulation of active dendritic channels. If they do play such a role in regulating intrinsic dynamics of a neuron, what is the spatial impact of such regulation? Specifically, does glial activity induce localized intrinsic plasticity or is the impact of glia-mediated intrinsic plasticity widespread thereby acting as a global regulator of neuronal computation and output? Answering these questions would require a systematic experimental approach with direct electrophysiological measurements spanning various dendritic and somatic locations. An important consideration while assessing the impact of gliotransmission on neuronal physiology is the observation that there is a common set of transmitter molecules (e.g., glutamate and GABA) that can be released by either neurons or glia. This calls for the experimental strategies where the release of these molecules can be controlled precisely from the glial cells to avoid interpretational ambiguities in their cellular sources. Recent advances in the optogenetic manipulation of astrocytes [273, 274] and chemogenetic strategies, where designer receptors exclusively activated by designer drugs (DREADD) [275] are specifically targeted on the glial cells, could provide reliable solutions towards realization of this objective.

Glial cells are also involved in several other forms of non-synaptic plasticity in neurons. For instance, myelination of axons by the myelinating oligodendrocyte can undergo activity-dependent changes. Specifically, increase in the performance of cognitive task and learning and memory has been shown to be associated with increased myelination in several model systems. Additionally, axonal myelination is dependent on the electrical activity of the axons where increase in the electrical activity induces more myelination of these axons and vice versa. Additionally, emerging lines of evidence show that myelination is an outcome of elaborate activity-dependent signaling among the perinodal astrocytes, oligodendrocytes, and the axons [276–281]. Any imbalance in such interactions can lead to pathological disorders such as the demyelination disorders that can manifest in several ways. Notably, pathological plasticity and excitotoxicity in the oligodendrocyte is a major cause of demyelination diseases [282, 283]. Finally, pathological plasticity in astrocytes can result in the imbalance of ionic and glutamate homeostasis aggravating epilepsy [284–287] and ischemia associated neuronal death [288–290]. Together, interactions between active glial signaling and signaling cascades that alter active dendritic mechanisms should further expand on this extensive literature, specifically providing direct clues on glial regulation of location-dependent input processing in neurons.

## Future Directions: Probing the Breadth and Depth of Subcellular Interactions

It is now evident that the constituent channels and components present on the ER and the plasma membrane interact in several ways to shape neuronal physiology—many of which have been investigated before and are discussed in this review. In light of these findings, and especially given the abundance and diversity of receptors, ion channels, pumps, and scaffolding molecules expressed on the neuronal membrane, there could be a myriad of ways through which these molecules interact with similar components present on the ER membrane. Thus, the ER membrane-plasma membrane interactions uncovered so far constitute a small subset of a large class of interactions between the two membranes. For instance, apart from the role of KA channels discussed above, several other VGICs could alter the influx of calcium into the cytosol and hence regulate the ER calcium release and consequent spread of calcium waves in the neurons. Some of these channels mediate direct influx of calcium, while others play critical roles in regulating membrane excitability thereby altering the extent of membrane depolarization-induced calcium entry. Given the complex kinetics and voltage-dependent gating profiles of these VGICs that differ significantly from one type to another and differences in their expression densities within and across neurons, it is expected that their contribution towards the regulation of ER calcium release would be non-trivial, differential, and variable.

Thus, a systematic analysis of the impact of various VGICs in regulation of emergence and spread of calcium waves is essential to decode this complex network of channels, receptors, and their spatiotemporal extent. For instance, calcium waves can be initiated at specific neuronal compartments (say an oblique dendritic structure) and simultaneous localized pharmacological blockage of various VGICs can shed light on their role(s) in regulating the spatiotemporal spread of calcium waves. Similarly, calcium released from the ER store can activate various downstream signaling pathways which could have varied molecular targets located on the plasma membrane. We have discussed that the flux of calcium through InsP_3_Rs is both necessary as well as sufficient to induce plasticity of HCN channels present on the plasma membrane. Future studies could explore the casualty of such ER calcium driven signaling pathway in the regulation of other VGICs, receptors, pumps, and other transmembrane and cytosolic proteins and enzymes.

## Future Directions: Probing the Breadth and Depth of Glia-Dendrite Interactions

Neuronal dendrites can release retrograde messengers upon post-synaptic depolarization [291, 292]. Do dendritic SEPs that constitute large dendritic depolarizations also translate into the release of retrograde messengers from the dendrites? How local is such a release, and how do they alter presynaptic neuronal terminals and their release properties? As astrocytic membranes are also endowed with the receptors for endocannabinoid whose activation plays a critical role in regulating synaptic plasticity and gliotransmission [214, 225], would such SEP-activated neuronal retrograde messenger release act as a complex feedback loop that further tightens astrocyte-neuron interactions? Given the diversity of retrograde messengers [291, 292], it is important to investigate if there are differences in the dendritic regulation of astrocytic activity with respect to the retrograde messengers they release. For instance, it would be interesting to ask if such differential release of various retrograde messengers differentially regulates gliotransmission.

What are the neuronal mechanisms that translate into higher impact of gliotransmission in the distal dendritic compartments as evident from the emergence of larger amplitude SEPs at these locations? In the case of synaptic scaling, the higher amplitude of EPSPs in the distal dendrites is attributed to higher densities of AMPA receptors in the dendrites [293–295]. Notably, two different subtypes of extrasynaptic NMDARs mediate SEPs [242]. Is there a density gradient of these receptors that lead to the higher amplitude SEPs in the dendrites? Or is the manifestation of such phenomenon a reflection of higher excitability of distally located astrocytes, thereby resulting in higher gliotransmission there? Addressing these questions is central towards understanding the compartmentalized vs. global nature of astrocyte-neuron interactions. Finally, differences in the dorsoventral population of neurons in the hippocampus in terms of their intrinsic properties, connectivity profiles, and neuronal plasticity are well established [182, 296–302]. Additionally, there are significant differences between superficial and deep pyramidal neurons in terms of afferent inhibition, channel properties, physiological characteristics, and morphology [303–306]. Against this background, it is important to ask whether there are differences in the impact of gliotransmission and neuron-astrocyte interactions between the dorsal and the ventral hippocampus and between superficial and deep neurons. Such investigations, involving direct paired astrocytic and somato-dendritic recordings along the dorsoventral and deep-superficial axes would shed further light on nuanced interactions among neurons and astrocytes and reveal the presence of any gradients in such interactions.

## Future Directions: Plasticity in Subcellular and Interactional Mechanisms Across Neurons and Glia

How plastic are the properties of the ER and its receptors? Experimental studies have shown that the morphological organization of the ER store is highly dynamic and exhibits activity-dependent remodeling. For instance, rise in the cytosolic calcium concentration can result in reversible fragmentation of the ER tubules. Additionally, the ER membrane can migrate towards the plasma membrane to form functional SOC channels [2, 159, 307]. Given such structural regulation of the ER membrane, can their receptors and channels, both on astrocytes and neurons, also undergo activity-dependent changes in their density and distribution as a consequence of calcium through the several calcium sources in these structures? If yes, is the nature of such plasticity on the impact of ER mediated signaling spatially localized or widespread throughout the neuron/astrocyte?

Much of our understanding about the functioning of the brain is based on the studies that illustrate the properties and plasticity of neurons. Comparatively speaking, our lack of understanding about the nuances of glial function and especially plasticity is rather astonishing. We know very little about the mechanisms and scope of glial plasticity in the CNS. Although emerging literature has focused on the impact of glia on neuronal synaptic plasticity, whether there are congruent long-term and/or short-term changes in the glial physiology and constitutive components largely remains to be explored. Although some studies have reported activity-dependent changes in the morphology of astrocytes and microglia but we have very limited understanding about the activity-dependent plasticity of various ion channels, receptors and transporters that critically regulate the physiology of these cells [308–312].

Astrocytic calcium signaling exhibits activity-dependent changes in terms of calcium spatiotemporal dynamics following synaptic activation [201, 313]. Thus, one can also ask whether changes in the glial calcium signaling is dependent on the amount of calcium present in ER store or on changes to the release machinery therein or on other calcium sources that support calcium elevation within glial cells? In other words, what are the consequences of the depletion of the ER calcium stores on glial physiology and how does it compare with the consequences of the neuronal ER store depletion? Understanding the cellular mechanisms and plasticity rules that govern these changes in the astrocytic function and their interactions with the neuronal plasticity rules would be a big step forward towards our understanding of glial physiology and neuron-glia interactions.

Astrocytes form highly interconnected networks in which they communicate freely with each other via passages of molecules and ions through gap junctions. Thus, they are ideally placed to integrate and regulate the flow of information in the neuron-glial circuit. Thus, one can ask if there are differences in the glial regulation of such information flow in the brain. For instance, is the flow of information in the astrocytic syncytium also plastic and what are the consequences of such plasticity towards information processing and storage in the brain? A recent study revealed input-driven changes in gap junction-dependent coupling in the astrocytic syncytium in the trigeminal main sensory nucleus [253]. There, it was shown that such input-driven changes in gap junctional coupling regulate rhythmic firing in neurons. Future studies could investigate the roles of such input-driven changes in gap junctional coupling in other brain regions, apart from assessing the possibility on whether activity-dependent changes in astrocytic connexin/pannexin density could regulate gliotransmission and astrocyte-dendrite interactions across different brain regions.

The advent of new technologies has continuously advanced our understanding of neuronal and glial physiology. State-of-the-art investigation techniques can be employed to answer some of these outstanding questions regarding glial physiology and plasticity. For instance, with the help of super resolution microscopy [314, 315], it is now possible to track the fate of a single molecule over time in live tissues. This can be harnessed to study the expression profiles and changes in the surface expression of various receptors expressed on the glial membrane. Combining this technique with presentation of activity patterns that alter functional and morphological properties of astrocytes can be used to investigate plasticity of receptors, channels, and transporters on the astrocytic membrane. Furthermore optogenetic and chemogenetic activation of astrocytes are being increasingly used for specific activation of glial signaling pathways and provide powerful tools to alter glial activity in vivo [316, 317]. It is an exciting time to study dendrites and glial cells—two structures that were falsely relegated to be passive nutrient suppliers—given the availability of these new techniques and the several unanswered, yet critical questions associated with these two structures and their interactions.
